# DISCLOSE : DISsection of CLusters Obtained by SEries of transcriptome data using functional annotations and putative transcription factor binding sites

**DOI:** 10.1186/1471-2105-9-535

**Published:** 2008-12-16

**Authors:** Evert-Jan Blom, Sacha AFT van Hijum, Klaas J Hofstede, Remko Silvis, Jos BTM Roerdink, Oscar P Kuipers

**Affiliations:** 1 Molecular Genetics, Groningen Biomolecular Sciences and Biotechnology Institute, University of Groningen, The Netherlands; 2 Interfacultary Institute for Genetics and Functional Genomics, Ernst-Moritz-Arndt-University, Friedrich-Ludwig-Jahnstraße, 15A 17487, Greifswald 17489, Germany; 3 NIZO Food Research, PO Box 20, 6710 BA Ede, the Netherlands; 4 Institute for Mathematics and Computing Science, University of Groningen, Nijenborgh 9, 9747 AG, Groningen, The Netherlands

## Abstract

**Background:**

A typical step in the analysis of gene expression data is the determination of clusters of genes that exhibit similar expression patterns. Researchers are confronted with the seemingly arbitrary choice between numerous algorithms to perform cluster analysis.

**Results:**

We developed an exploratory application that benchmarks the results of clustering methods using functional annotations. In addition, a *de novo *DNA motif discovery algorithm is integrated in our program which identifies overrepresented DNA binding sites in the upstream DNA sequences of genes from the clusters that are indicative of sites of transcriptional control. The performance of our program was evaluated by comparing the original results of a time course experiment with the findings of our application.

**Conclusion:**

DISCLOSE assists researchers in the prokaryotic research community in systematically evaluating results of the application of a range of clustering algorithms to transcriptome data. Different performance measures allow to quickly and comprehensively determine the best suited clustering approach for a given dataset.

## 1 Background

DNA microarray technology is commonly used to study mRNA expression levels of genes under different experimental conditions. Clustering approaches are widely used in the analysis of gene expression data. The ability to identify groups of genes exhibiting similar expression patterns by clustering allows for detailed biological insights into global regulation of gene expression and cellular processes. Clustering methodology is considered a potent means to infer putative gene function [1,2].

In the process of the analysis of transcriptome data, researchers are often faced with the choice between a wide variety of clustering methods and associated parameters. The results of the application of different clustering algorithms to the same dataset will place genes in different clusters and therefore result in different biological interpretations of the same dataset. Moreover, selecting the most appropriate clustering method and parameters heavily depends on the experience of the researcher and on the nature of the dataset analyzed.

Several studies have shown the relevance of applying external measures (i.e., using prior biological knowledge) to more objectively evaluate the results of clustering algorithms ([3-6]). Central in this approach is the assumption that genes involved in similar biological processes are more likely to be co-transcribed. Therefore, selecting a clustering method the clusters of which are most enriched with biological processes is considered as a relevant starting point for the biological interpretation of a DNA microarray dataset [6-9].

Co-clustered genes may also represent a candidate set of coregulated genes, i.e., genes of which the expression is regulated by the same transcription factor. The discovery of putative regulatory motifs in *cis*-regulatory regions of genes that are part of the same cluster could therefore allow identification of new TF targets [10]. Existing implementations that employ motif discovery on clusters obtained by DNA microarray [7,8,11] leave the downstream analysis of the motifs to be performed by the researcher. More importantly, no feedback concerning the results of the analysis is presented for the used clustering algorithm and associated parameters, making it difficult to compare the effect on the results of different clustering parameters or methods to the same dataset. Ideally, quantitative information concerning the functional and motif enrichments of the tested clusters should be provided after each clustering analysis. This information would then allow for a more objective selection of optimal clustering parameters based on biological criteria. Lastly, all available software packages are not specifically suited for prokaryotic data analysis since they do not support prokaryote-specific data sources (e.g., operons, specific genome annotations).

We have developed the application DISCLOSE for prokaryotes that benchmarks clustering methods using biological annotations and the SCOPE DNA binding site detection algorithm [12]. This algorithm allows the prediction of *cis*-regulatory motifs of genes which are part of the same cluster. In addition, additional occurrences of identified motifs are determined. Moreover, putative motifs are compared with known DNA binding sites as well as a functional analysis of the genes bearing the motif in their upstream region.

## 2 Program overview

The DISCLOSE application allows for an automated scoring based on different criteria of the different clusters in each clustering analysis. This scoring is followed by a decision by the researcher on the most suitable clustering method for the dataset analyzed based on one metric. Various metrics (see below) are available to assess the results of the clustering analysis. Each metric provides for a unique measure to filter the results of a clustering analysis and can therefore be used to address different research questions; e.g., selection of a clustering analysis which yields a large number of overrepresented motifs or a clustering analysis which produces a large number of significant overrepresented metabolic pathways. Based on the chosen clustering analysis, DISCLOSE provides an in-depth analysis of clustering results together with an intuitive visualization.

### 2.1 Input

**Figure 1:**
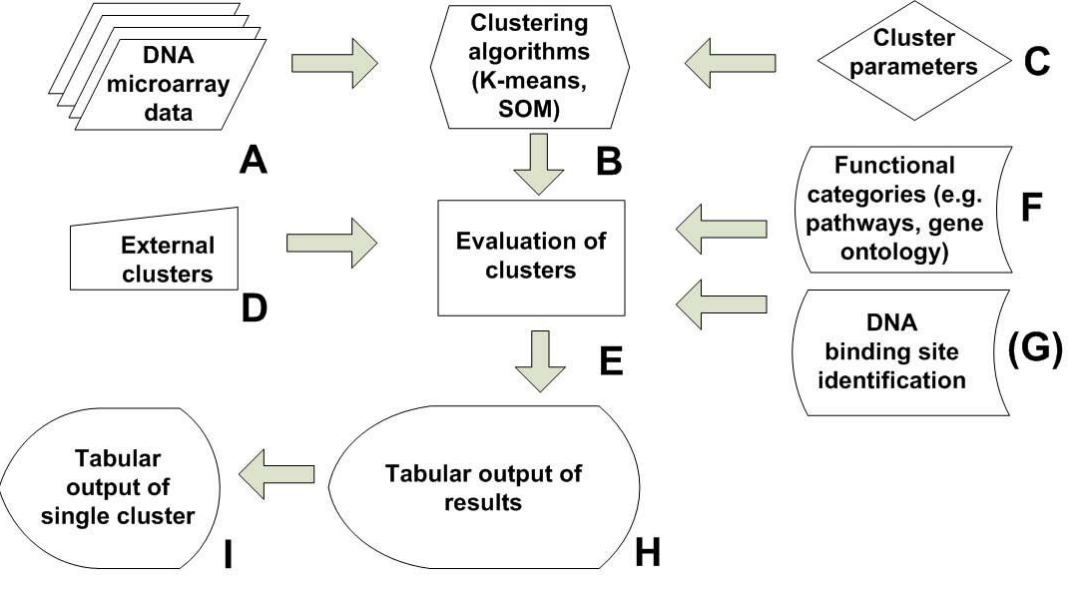
**Flow diagram**. The DISCLOSE application uses functional categories to evaluate the cluster results given a dataset (A), clustering algorithms (B), and clustering parameters (C). Clustering can be performed by the DISCLOSE application or based on results from external clustering programs (D). Each clustering run is evaluated (E) for overrepresented functional categories by the program using different annotation sources (F), and optionally by a motif identification algorithm (G). Lastly, results of the clustering analysis are cumulated in a tabular display in which each row shows the summary of the application of a clustering method to the data (H). From the tabular display, selecting results for an individual clustering (I) allows for a cluster based analysis (see Fig. 2A).

A process overview of DISCLOSE is shown in Figure 1. The input data for DISCLOSE consists of transcriptome data (Fig. 1A) and genome files (e.g., EMBL or Genbank). DISCLOSE supports a broad variety of prokaryotic gene identifiers, including locus tags and gene names.

The DNA binding site detection algorithm uses operon information and the genomic sequence in FASTA format (see Fig. 1G). However, DISCLOSE also supports a single gene-based analysis if no operon information is available. Moreover, known binding site information can be used to evaluate the results of the DNA binding site detection algorithm.

### 2.2 Processing

#### 2.2.1 Clustering of gene expression data

Two widely used clustering algorithms (K-means and Self Organizing Maps) from the TIGR Multiexperiment Viewer (MeV) package  are implemented in DISCLOSE (Fig. 1B). The application of different parameter settings to a clustering analysis is facilitated by allowing for a parameter range and/or different correlation measures for each clustering approach, e.g., a K-means clustering with five to twenty clusters using Euclidean and Pearson correlation measures (Fig. 1C).

#### 2.2.2 Evaluation of external clustering applications

The two most used clustering algorithms have been implemented in DISCLOSE. Since many other clustering methods exist, we have chosen to allow the evaluation of results obtained from external clustering applications in DISCLOSE (Fig. 1D).

#### 2.2.3 Evaluating clusters using biological knowledge

The identification of significantly enriched categories in a cluster of co-expressed genes enables users to focus on relevant biological phenomena. Assuming a normal distribution of the number of genes from the clusters for each functional category, one expects a difference in the proportion of genes for a category present in each cluster compared to the genes from a reference set (e.g., the remaining genes from the studied organism). To identify clusters that contain a significantly enriched number of genes from a certain functional class, the distribution of genes from a gene set (e.g., a cluster of genes) is compared to the genes in the reference set (e.g., the remainder of the genes in all other clusters).

A hypergeometric distribution test is used to calculate *p*-values for each functional category from each cluster. This *p*-value describes the probability of observing an enrichment of genes from a functional category in a cluster by chance (Fig. 1E). The number of false-positives for the initial cluster evaluation (Fig. 1E) is controlled by a strict Bonferroni multiple testing correction (taking into account the clustering runs), while additional corrections ([13]) are used upon detailed analysis of selected results.

#### 2.2.4 De novo identification of DNA binding sites

Clustering algorithms allow for the identification of groups of genes that exhibit similar expression patterns. This co-expression could be explained by transcriptional co-regulation. Identification of overrepresented DNA binding sites in genes of the same cluster is performed by the SCOPE method [12]. This method utilizes three specialized algorithms; BEAM for non-degenerate motifs, PRISM for degenerate motifs and SPACER for bipartite motifs. Key aspects of SCOPE are high sensitivity and specificity for a broad range of motifs (i.e., perfect, degenerated and gapped motifs), requirement of a minimum of parameters for motif detection, and speed [12].

#### 2.2.5 Characterization of putative motifs

For various model organisms reference databases on transcriptional regulation have been created that summarize experimentally characterized transcription factors, their binding sites and the genes they regulate (e.g., from DBTBS [14] for *Bacillus subtilis *or regulonDB [15] for *Escherichia coli*). The binding sites derived from these databases can be incorporated in DISCLOSE, allowing for a comparison of known binding sites to putative DNA binding sites found by the SCOPE algorithm. This feature allows researchers to distinguish between known and unknown binding sites. In addition, aligned putative motif instances identified by the SCOPE algorithm are used to create position specific scoring matrices. These matrices are subsequently used to score the upstream and coding DNA sequences from all genes of the studied organism. The prioritized results of this analysis allow researchers to identify additional genes that do contain the putative motif but were not part of the original cluster.

Lastly, DISCLOSE attempts to functionally characterize motifs by identifying significantly enriched categories using the genes that contain the motif. This analysis is different as compared to a standard functional enrichment analysis. Since the motif analysis only uses operon information, the enrichment of categories is calculated by taking into account the operons instead of genes. This analysis yields a *p*-value which describes the probability of observing an enrichment of operons belonging to a specific functional category in the operon members bearing the motif by chance. The results of this examination yield insights concerning the biological processes that could be controlled by the putative motif.

### 2.3 Output

Quantitative results of over representations for each clustering evaluation (e.g., a K-means clustering for 20 clusters using a Euclidean correlation measure) are represented in a tabulated view (Fig. 1I). A single row in this view includes information from an individual clustering run for the following metrics:

1. Number of significant overrepresented functional categories from each annotation source (e.g., the total number of overrepresented metabolic pathways).

2. Total number of significant overrepresented functional categories from all annotation sources (e.g., all overrepresented metabolic pathways, GO categories etc).

3. The number of clusters which are enriched for one or more functional categories.

4. The score of the most overrepresented DNA binding site.

5. The number of overrepresented DNA binding sites that exceed a predefined threshold.

6. Number of functional categories from one annotation source that were found overrepresented in gene members of a cluster that contain a certain motif in their upstream region.

7. Number of functional categories for all annotation sources that were found overrepresented in gene members of a cluster that contain a certain motif in their upstream region.

Several filtering options are available for each of the described metrics. Each metric provides for a unique measure for users to select the highest scoring clustering results based on different criteria, e.g., a clustering that yields the highest number of overrepresented metabolic pathways or the most significantly overrepresented DNA binding sites. This allows researchers to select the most optimal clustering run for their research question. Graphical representations of the results are available for different stages in the analysis which will be discussed in the upcoming sections.

#### 2.3.1 Complete results analysis

In addition to saving the complete contents of the tabulated view to a HTML file, a robustness analysis is performed on all of the clustering runs. This analysis determines the frequency of occurrence for every functional category (e.g., a robustness frequency for a functional category of 50% indicates that it is significantly overrepresented in individual clusters from 50% of the clustering runs). Robust functional categories are a good starting point for an analysis since they occur in relatively large fractions of the clustering results. Less robust functional categories allow for the analysis of functional categories that would have been missed using more general clustering parameters.

#### 2.3.2 Functional analysis for individual clustering runs

**Figure 2:**
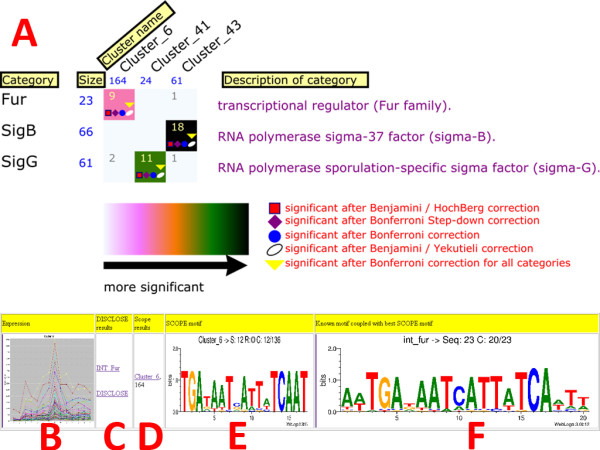
**Visualization**. A. Genes from the DNA microarray data were clustered. The size of each cluster is displayed in blue underneath the cluster name. Numbers in each colored rectangle represent absolute values of occurrences. The significance of the overrepresentation is visualized in a colour gradient which is displayed at the bottom of the plot. The description of each category is placed at the right. Multiple testing correction results are visualized using five different symbols to distinguish between the individual corrections. The number of symbols placed in each rectangle corresponds to the number of multiple testing corrections after which the annotation is found significant (see [13] for more details concerning this visualization). The graphical representation of the overrepresented DNA binding sites from the SCOPE algorithm consists of several components. The results of SCOPE based on a single cluster are discussed: B. The expression graph of the genes in the cluster. C. Contains information concerning overrepresented functional categories and a link to the results of DISCLOSE. D. Link to the results of SCOPE. E. The highest scoring motif found in the cluster. F. The highest scoring motif is compared with existing binding site information. The known motif that matches the putative motif best is displayed.

Based on the results of a single clustering run, a graphical representation (Fig. 2A) of the overrepresented categories is generated by FIVA [13]. Individual cluster information from a single clustering run is available, which enables a focused analysis of individual clusters.

#### 2.3.3 Visualization of putative DNA binding sites for individual clustering runs

**Figure 3:**
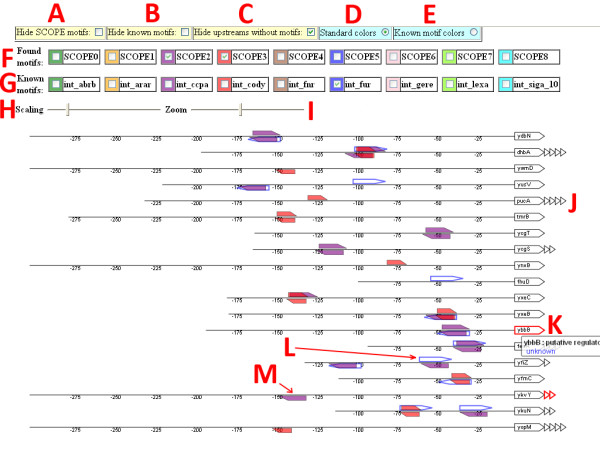
**A customizable graphical representation of DNA binding sites**. The Scalable Vector Graphics visualization displays the genomic context of putative and known motifs in the upstream sequences of the operons. The user interface allows users to interact with the visualization. A) Hide *de novo *motifs. B) Hide known motifs from literature. C). Hide upstream regions without any putative or known motifs. D). Use standard coloring of putative motifs. E). Use coloring of putative motifs based on best hit with known motif. F). Every found motif can be displayed or hidden from the visualization using checkboxes. G) Known motifs can be displayed or hidden from the visualization using checkboxes. H). The scaling slider adjusts the width between the upstream sequences. I). The zooming slider allows for zooming of the visualization. J). The first structural gene of each operon is a large polygon, whilst the other genes are represented using smaller polygons. K). Genes coding for a putative regulator are colored red. Hovering with the mouse over the genes creates a tooltip displaying the function of the gene. L). Open polygons represent known binding sites derived from literature sources. M). Filled polygons depict putative motifs.

In addition to the functional analysis described in the previous section, DISCLOSE is capable of identifying overrepresented DNA binding sites in clusters of co-expressed genes. The putative DNA binding sites that are identified from the SCOPE algorithm are visualized as sequence logos [16] and displayed in HTML files (Fig. 2A to Fig 2F). In addition, putative motifs are compared with known DNA binding site information (e.g., from DBTBS [14] or regulonDB [15], see Fig. 2F). The results of this comparison are integrated in the HTML files. Detailed information for every putative motif is available, showing identified functional enrichments of in the operon members of the motif and the raw output of the SCOPE algorithm in text files. Moreover, information concerning additional occurrences of the putative motif in the upstream or coding regions from other operons of the genome is integrated in the HTML files. Lastly, an operon visualization is created which show the genomic context for all known and putative motifs to facilitate the motif analysis (Fig 3).

## Results and discussion

Our DISCLOSE application was used to identify significant functional categories and DNA binding sites. To evaluate the performance of our program, we compared the original clustering analysis results (see Table 1) of a time course DNA microarray experiment of *B. subtilis *([17]) with the findings of DISCLOSE. With this time-course experiments the authors explore the transcriptional changes that occur during germination and outgrowth of *B. subtilis *spores. The highest scoring categories from our DISCLOSE analysis, i.e., categories with a significance frequency above 10%, are listed in Table 2. Overlapping redundant categories that represent similar functional groupings were removed from the table. The majority of the significantly overrepresented categories that were identified by our analysis recapitulated the original results.

**Table 1:** Biological phenomena discussed in the original article

Functional category	DISCLOSE	Significance frequency
Purine biosynthesis	X	91%

Cell growth	X	88%

General stress response	X	88%

tricarboxylic acid cycle	X	86%

Sigma D regulon (motility)	X	85%

Glycolysis	X	72%

cell division	X	71%

pyrimidine biosynthesis	X	70%

DNA replication and DNA repair functions	X	66%

Sulfur amino acid metabolism	X	19%

aspartate metabolism	X	16%

serine metabolism	X	12%

fatty acid biosynthesis	X	12%

drug transporter activity	X	3.1%

Na+/H+ antiporters	X	0.6%

RNA modification	X	0.3%

Multidrug transporters	-	

The original analysis of the study of Keyser *et al *[17] revealed several biological phenomena that are found to be induced during the DNA timecourse experiment. The described biological phenomena were matched with the results of the robustness analysis (complete analysis is listed in Table 2). Phenomena discussed in the original analysis are listed in the first column. A match with the results of DISCLOSE is indicated in the second column. Information concerning the significance frequency is shown in column three.

**Table 2:** Results of robustness analysis of DISCLOSE

Functional category	Member size	Significance frequency	In original study
GO-0006164 : purine nucleotide biosynthetic process	28	94.03%	Y

COG-F : Nucleotide transport and metabolism	84	93.56%	Y

GO-0003735 : structural constituent of ribosome	59	88.06%	Y

INT-SigB : general stress sigma factor	66	87.91%	Y

COG-J : Translation, ribosomal structure and biogenesis	161	87.12%	Y

PW-path-bsu00020 : Citrate cycle	18	86.18%	N

GO-0003723 : RNA binding	107	85.87%	Y

COG-N : Cell motility and secretion	57	85.71%	Y

INT-SigG : late forespore-specific gene expression	61	84.30%	N

PW-path-bsu00193 : ATP synthesis	8	83.04%	Y

UP-67 : Ligase	78	79.74%	Y

GO-0006935 : chemotaxis	26	73.46%	Y

UP-56 : Glycolysis	19	72.21%	Y

UP-29 : Cell division	32	71.11%	Y

PW-path-bsu00720 : Reductive carboxylate cycle	13	70.32%	N

PW-path-bsu00240 : Pyrimidine metabolism	51	68.60%	Y

PW-path-bsu00970 : Aminoacyl-tRNA biosynthesis	23	68.28%	Y

COG-L : DNA replication, recombination and repair	138	65.93%	Y

UP-15 : Threonine biosynthesis	3	58.55%	N

COG-G : Carbohydrate transport and metabolism	246	55.88%	Y

GO-0006520 : amino acid metabolic process	184	53.53%	Y

UP-124 : Sporulation	180	49.92%	Y

INT-SigK : late mother cell-specific gene expression	57	47.40%	N

PW-path-bsu03070 : Type III secretion system	9	44.27%	N

INT-PurR : negative regulation of the purine operons	10	43.32%	N

GO-0015293 : symporter activity	82	35.63%	Y

UP-25 : Porphyrin biosynthesis	13	27.62%	N

UP-179 : Folate biosynthesis	6	27.31%	N

PW-path-bsu00190 : Oxidative phosphorylation	31	26.05%	N

PW-path-bsu02060 : Phosphotransferase system (PTS)	27	24.96%	N

COG-D : Cell division and chromosome partitioning	33	24.64%	Y

GO-0008360 : regulation of cell shape	36	24.17%	Y

PW-path-bsu00740 : Ribo avin metabolism	5	23.54%	N

PW-path-bsu00030 : Pentose phosphate pathway	24	23.39%	N

GO-0009086 : methionine biosynthetic process	15	22.76%	Y

UP-17 : Hydrogen ion transport	15	21.66%	Y

INT-SigE : early mother cell-specific gene expression	82	21.66%	N

PW-path-bsu00920 : Sulfur metabolism	15	19.62%	N

INT-SigA : RNA polymerase major sigma-43 factor	320	18.52%	N

COG-O : Posttranslational modification, protein turnover, chaperones	98	17.11%	N

PW-path-bsu00400 : Phenylalanine, tyrosine and tryptophan biosynthesis	28	16.16%	N

PW-path-bsu00252 : Alanine and aspartate metabolism	21	16.01%	Y

GO-0009252 : peptidoglycan biosynthetic process	32	15.22%	Y

PW-path-bsu00260 : Glycine, serine and threonine metabolism	34	12.55%	Y

UP-84 : Fatty acid biosynthesis	11	12.55%	Y

GO-0000103 : sulfate assimilation	7	10.67%	N

GO-0000105 : histidine biosynthetic process	11	10.36%	N

PW-path-bsu00670 : One carbon pool by folate	11	10.20%	N

The performance of DISCLOSE was evaluated by comparing the clustering analysis results of a time course DNA microarray experiment of *Bacillus subtilis *([17]) with the results obtained by DISCLOSE. This analysis recapitulated most of the findings of the original study. In addition, several significantly overrepresented categories were found by DISCLOSE that were not discussed by the authors. The following table list the functional categories that are identified by DISCLOSE from the robustness analysis with a significance frequency of at least 10%. The last column indicates if a category was found significant in the original study. Redundant functional categories were removed from the table (e.g., functional category GO:0006096-glycolysis was not listed in the table since this functional category was already covered by a UniProt category UP:56-glycolysis). (Abbreviations used; GO : Gene Ontology, INT : Regulon, PW: metabolic pathway, UP : UniProt and COG : Clusters of Orthologous Gtroups)

Furthermore, additional biological phenomena were identified by DISCLOSE that were not discussed by the authors (Table 2). However, some categories that were described in the original study did not meet the 10% threshold that was used in our analysis. These reported categories were not found in the original analysis using a clustering based analysis but using an analysis which has been performed on the highest expressed genes from every individual time point.

### DNA binding site analysis of DISCLOSE

**Figure 4:**
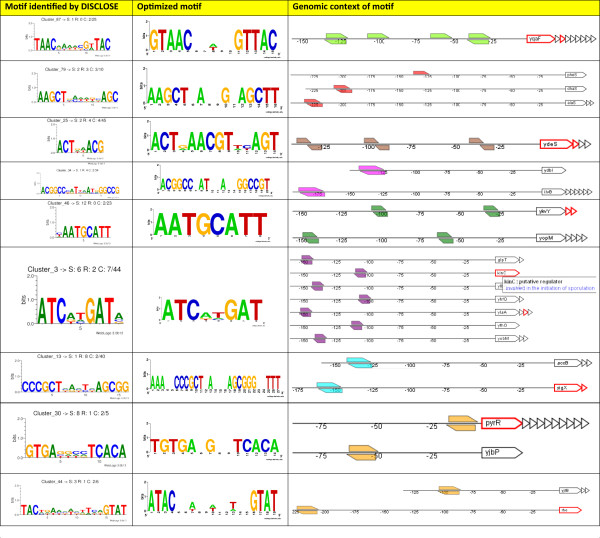
**Non-validated results of overrepresented DNA binding sites**. DISCLOSE was also able to detect several motifs in clusters that could not be matched with motifs from literature. The motifs identified by DISCLOSE are visualized as sequence logos [16] and are displayed in the first column. An optimized version of the motif is placed in the second column whilst the genomic context of the instances are displayed in column three.

For the overrepresented DNA binding site analysis of the data of Keijser *et al*, we selected the clustering run that yielded the highest number of overrepresented motifs. The combined visualization of known and putative motifs allowed for a rapid determination of genes with instances of motifs that matched to a known DNA binding site (see Additional file 1). A total number of 12 putative motifs that were identified by DISCLOSE matched one of the 45 motifs that are described in DBTBS. Additionally, 2 motifs were linked to known motif binding sites based on literature information. Furthermore, DISCLOSE also discovered a number of motifs that were not described before (Fig 4).

### Discussion of the results

The results of our DISCLOSE analysis show that we were able to identify most of the functional overrepresented categories that are discussed in the original study. However, a number of functional categories were not recovered using our approach due to the nature of the analysis employed in the original study which was based on analysis of differentially expressed genes in single time points. The single-time point analysis is tedious and does not take into account the temporal properties of the dataset.

The interactive visualization module for overrepresented motifs by DISCLOSE facilitated the detection of putative motifs as well as the discovery of motifs that have been described in literature.

## 3 Conclusion

Choosing a clustering method and associated parameters for a given DNA microarray dataset is a challenging task. Moreover, commonly used clustering algorithms lack the ability to annotate the clusters using functional information. This information is crucial to comprehend the underlying biology of the experiment. Here, we present DISCLOSE, an exploratory application that benchmarks clustering methods using functional annotations and a *de novo *motif discovery algorithm. DISCLOSE allows to select the most appropriate clustering method and to visually inspect the clusters obtained for a given DNA microarray dataset. Our application quantitatively describes the most stable overrepresented functional categories in the clusters. This methodology allows for a more objective and complete interpretation of the dataset analyzed. Our application offers the following advantages to existing tools:

• benchmarks clustering methods using enrichment analysis and motif discovery

• supports K-means and SOM clustering algorithms

• robustness analysis for functional categories

• ready-to-use databases for over 600 prokaryotic organisms

• functional enrichment analysis of putative motifs

• identification of additional motif occurrences

• matching of putative motifs with known motifs

• interactive visualization of genomic context of known and putative motifs.

• stand-alone application (supports all major operating systems)

## Methods

### 4.1 Software package

DISCLOSE was programmed as a standalone application in Java using the Eclipse  framework and it runs on all Java-supporting operating systems (Windows, Linux and Mac OSX). The graphical output can be viewed by all web browsers that are able to process Scalable Vector Graphics.

DISCLOSE features the following annotation modules: i. Gene Ontology, ii. Metabolic pathways (KEGG), iii. COG classes, iv. Regulatory interactions, v. UniProt keywords vi. and user-defined functional categories (Fig. 1F). The supplementary materials contain ready-to-use databases for over 600 prokaryotic organisms. The software package contains in addition a manual and an example analysis using a publicly available dataset.

#### Dataset used for validation

The dataset is part of a transcriptome analysis from a study on the growth transitions of *Bacillus subtilis*. Data from this experiment was obtained from the Gene Expression Omnibus database from NCBI [18] (accession number: GSE6865). The authors of the original study applied a K-means clustering to reveal patterns of temporal gene expression. The optimum number of clusters was revealed by principal-component analysis and ordered by the timing of expression. A detailed analysis based on individual time points was conducted by JProGO [19] to identify overrepresented groups of functionally related genes. From this analysis, the authors have selected several functional categories from a list of significantly overrepresented categories (see Table 1).

#### DISCLOSE analysis

Our analysis was conducted using a K-means clustering using a range from 10 to 100 clusters for all correlation measures. For each cluster that was analyzed the DNA binding site reported the 10 most overrepresented motifs. Finally, the results of a clustering run that yielded the highest number of motifs with a score above 15 together with a robustness analysis with a 10% cut off for all clusters were analyzed.

#### Gene annotations

A genome file for *Bacillus subtilis *was obtained from NCBI and supplemented with db_xrefield information from an EMBL genome review file from EBI [20]. COG information from a local whog file [21] was loaded by using the organism abbreviation: bsu. Pathway information was obtained from KEGG [22]. The latest Gene Ontology (GO) obo file was used from the Gene Ontology website [23]. Functional categories based on Uniprot keywords [24] were imported as well as information from the DBTBS database [14] for the interaction annotation module.

## 4 Availability and requirements

• Project name: DISCLOSE

• Project homepage: 

• Supplementary information: 

• Operating systems: Microsoft Windows, Linux and Mac OSX

• Programming language: Java

• License: Freely available

• Any restrictions on use by non-academics: No

## Authors' contributions

EJB and SAFTH conceived the study. EJB programmed the program. RS and KJH designed and programmed the analysis interface. EJ and SAFTH drafted the manuscript. JBTMR and OPK guided and coordinated the project and were involved in correcting and improving the manuscript. All authors read and approved the final manuscript.

## Supplementary Material

Additional file 1 **Validated results of overrepresented DNA binding sites**. Various motifs that were identified by the DNA binding site identification module from DISCLOSE matched known motifs described in the literature. The motifs identified by DISCLOSE are visualized as sequence logos [16] and are displayed in the first column. The name of the matching regulon and the sequence logo based on aligned known motif instances are placed in the second and third column respectively. Click here for file
