# Supplementary figures and images for: DISCLOSE : DISsection of CLusters Obtained by SEries of transcriptome data using functional annotations and putative transcription factor binding sites
Source: BMC Bioinformatics. 2008 Dec 16;9:535. doi: 10.1186/1471-2105-9-535 (PMC2661003; doi:10.1186/1471-2105-9-535)

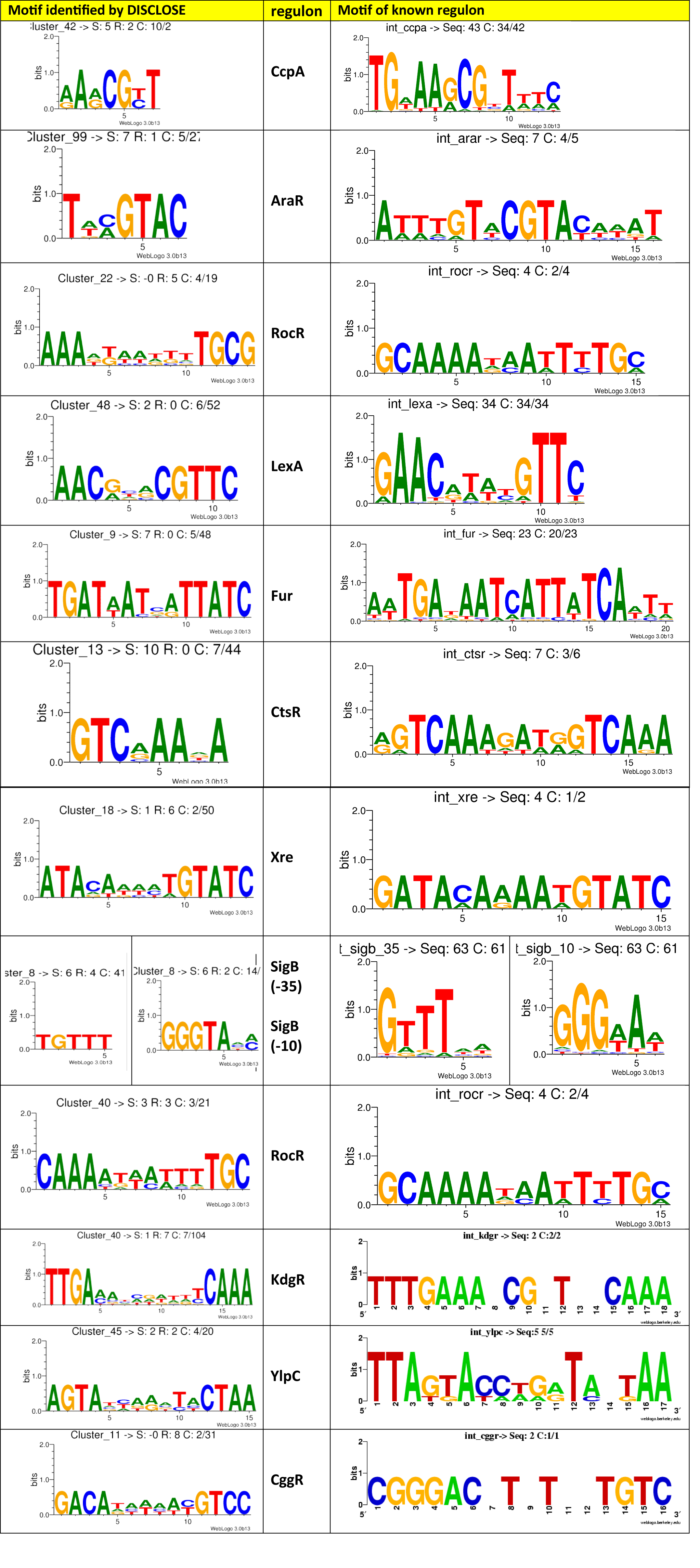

Supplement: Additional file 1 — Validated results of overrepresented DNA binding sites. Various motifs that were identified by the DNA binding site identification module from DISCLOSE matched known motifs described in the literature. The motifs identified by DISCLOSE are visualized as sequence logos [16] and are displayed in the first column. The name of the matching regulon and the sequence logo based on aligned known motif instances are placed in the second and third column respectively. [file 1471-2105-9-535-S1.png]
